# Elaboration of Functionalized Iron Oxide Nanoparticles by Microwave-Assisted Co-Precipitation: A New One-Step Method in Water

**DOI:** 10.3390/molecules29184484

**Published:** 2024-09-21

**Authors:** Thomas Girardet, Amel Cherraj, Pierre Venturini, Hervé Martinez, Jean-Charles Dupin, Franck Cleymand, Solenne Fleutot

**Affiliations:** 1 Institut Jean Lamour, UMR 7198, Université de Lorraine, 2 allée André Guinier, 54011 Nancy, France; thomas.girardet@univ-lorraine.fr (T.G.); amel.cherraj2@etu.univ-lorraine.fr (A.C.); pierre.venturini@univ-lorraine.fr (P.V.); franck.cleymand@univ-lorraine.fr (F.C.); 2 Institut des Sciences Analytiques et de Physicochimie pour l’Environnement et les Matériaux, UMR 5254, E2S UPPA, CNRS, IPREM, 64000 Pau, France; herve.martinez@univ-pau.fr (H.M.); jean-charles.dupin@univ-pau.fr (J.-C.D.); 3 Centrale Casablanca, Research Center for Complex Systems and Interactions, Bouskoura 27182, Morocco

**Keywords:** microwave process, coprecipitation, iron oxide, nanoparticles

## Abstract

Iron oxide nanoparticles are extensively utilized in various fields, particularly in biomedical applications. For such uses, nanoparticles must meet specific criteria, including precise size, morphology, physico-chemical properties, stability, and biocompatibility. Microwave-assisted co-precipitation offers an efficient method for producing water-soluble nanoparticles. Functionalization with citrate during synthesis is crucial for achieving a stable colloidal solution. This study aims to compare the effectiveness of conventional co-precipitation with microwave-assisted co-precipitation. The synthesized nanoparticles were characterized using TEM, DLS, FTIR, XRD, and magnetic measurements. The findings indicate that the in situ citrate functionalization during synthesis results in stable, non-aggregated nanoparticles.

## 1. Introduction

Nanomaterials are defined by their nanometer scale in at least one of the three spatial dimensions. Among these, nanoparticles (NPs) are unique in that all three dimensions are below 100 nm. A nanoparticle consists of an assembly of a few hundred to several thousand atoms, forming a nanometric object ranging from 1 to 100 nm. This exceptionally small size significantly increases the surface-to-volume ratio, which enhances their surface reactivity and consequently alters their intrinsic properties. Nanoparticles are increasingly used in various fields of research, such as pollution control [1,2], detection of heavy metals [3], energy storage [4], and catalysis [5].

In this field, iron oxide nanoparticles are particularly attractive due to the magnetic properties of magnetite (Fe_3_O_4_) and maghemite (γ-Fe_2_O_3_), which exhibit superparamagnetic behavior when below 20 nm, imparting them with ferrofluidic characteristics [6]. These magnetic properties imply that at room temperature, there is no spontaneous magnetism, which only emerges in the presence of an external magnetic field. This property is of great interest for various medical applications, including drug carriers [7], drug delivery systems [8], cancer therapy [9], hyperthermia [10], magnetic separation [11], and notably magnetic resonance imaging (MRI) [12]. However, several challenges must be addressed to enable the use of nanoparticles in the human body. The most critical of these include the precise control of physicochemical characteristics such as size [13], shape [14], composition [15], as well as ensuring the stability and prevention of nanoparticle aggregation over time [16]. 

Iron oxide nanoparticles can be synthesized through various methods, including hydrothermal synthesis [17], ultrasound [18], the sol–gel process [19], thermal decomposition [20], and co-precipitation [21]. Each synthesis method has its own advantages and disadvantages (Table 1). Co-precipitation was chosen for its high yield and simplicity in producing water-soluble nanoparticles at low reaction temperatures. However, this method also presents challenges, such as poor size control and low crystallinity. Conversely, thermal decomposition produces nanoparticles with excellent size control and high crystallinity, though the nanoparticles are stable only in organic solvents, necessitating a phase transfer step for biomedical applications [22]. 

In the classical co-precipitation, ferric salts (Fe^3+^) and ferrous salt (Fe^2+^) are precipitated in a basic aqueous medium while it is heated and stirred. The formula of the chemical reaction may be written as below [23]:

Fe^2+^_(aq)_ + 2 Fe^3+^_(aq)_ + 8 HO¯ _(aq)_ 🡪 Fe_3_O_4 (s)_ + 4 H_2_O. (1)

According to the reaction described, magnetite (Fe_3_O_4_) nanoparticles are theoretically produced, particularly when synthesis is conducted under a controlled atmosphere. Studies have shown that the composition of iron oxide nanoparticles is influenced by their size [23]. For particles smaller than 8 nm, the composition tends to favor maghemite, while for those larger than 12 nm, magnetite becomes the dominant phase. However, the surface of magnetite nanoparticles can oxidize, and for intermediate sizes, an oxidation gradient can be observed from the surface to the core, resulting in a composition that can be approximated as sub-stoichiometric magnetite (Fe_3−δ_O_4_) [23]. The crystal lattice of magnetite is an inverse spinel structure, which transitions to maghemite upon air oxidation. This transition creates vacancies within the inverse spinel structure, leading to different possible states of organization [6]. 

Nanoparticles synthesized through the above reaction are uncoated and tend to aggregate, causing them to lose stability in the solvent—typically water in the co-precipitation process. To address this issue, nanoparticles can be functionalized with ligands such as oleic acid [24,25], oleylamine [26], polymers such as PVA, PVP [27,28] or citric acid [29,30]. The choice of ligand is crucial for ensuring stability in the solvent. For instance, oleic acid and oleylamine provide stability in organic solvents, whereas citric acid and polyacrylic acid are water stable. Given the goal of synthesizing nanoparticles for potential biomedical applications and their use in the human body, citric acid was selected for its biocompatibility [31]. Indeed, citric acid is a component of the Krebs cycle, present in every cell of the human body [32]. Ligand functionalization can be performed either during or after the synthesis of iron oxide nanoparticles, which helps reduce particle size and, by extension, improves their stability. 

Co-precipitation is a chemical process first described by the LaMer model in the 1950s [33]. It involves four distinct steps to form nanoparticles: inorganic polycondensation, nucleation, growth, and Ostwald ripening [34]. Various reaction parameters influence nanoparticle formation, including the type of ferric salts, the Fe^2+^/Fe^3+^ ratio, temperature, pH value, ionic concentration of the reactants, and the nature of the base used. 

The conventional co-precipitation process was adapted for microwave use through the development of a new protocol. Since the 2000s, microwave processes have been increasingly employed to reduce synthesis time and increase yield [35]. Microwave irradiation causes the solvent to vibrate, thereby heating the solution uniformly and reducing the size distribution of the nanoparticles [36]. As mentioned earlier, the size of nanoparticles is dependent on the temperature of the solution; any temperature gradients can lead to a broad size distribution. This study aims to compare nanoparticles synthesized via conventional co-precipitation with those obtained through a microwave-assisted process. 

Microwaves are a form of heating device that uses microwave irradiation, with wavelengths ranging from 1 mm to 1 m. They enable dielectric heating through two primary mechanisms: dipolar polarization and ionic conduction [36]. Dipolar polarization relies on the alignment of dipole molecules such as water with the microwave field, causing them to rotate and generate heat through friction. Ionic conduction, on the other hand, involves ionic components like Fe^2+^ and Fe^3+^, which align and oscillate under the microwave field. This oscillation leads to increased collisions between particles, further generating heat. 

Microwave heating is more efficient, ensuring that more precursor molecules reach the optimal temperature for reaction. This enhances the nucleation rate, thereby improving the overall yield of the reaction. Numerous studies have compared the classical synthesis of iron oxide nanoparticles with microwave-assisted methods [37,38,39]. However, these studies often compare thermal decomposition with microwave processes. In contrast, for conventional co-precipitation, the resulting nanoparticles are not optimized and tend to aggregate rapidly. In this study, we aim to compare conventional co-precipitation with a modified approach (using a ligand) under the same microwave-assisted protocol.

To evaluate the efficiency of the synthesis, physicochemical and magnetic characterizations of the obtained nanoparticles were systematically conducted using standard methods: Transmission Electron Microscopy (TEM), Dynamic Light Scattering (DLS), Fourier Transform Infrared Spectroscopy (FTIR), X-ray Diffraction (XRD), and Thermogravimetric Analysis (TGA). Magnetic measurements were performed using a SQUID VSM to determine the magnetic state and properties of the nanoparticles.

## 2. Results

In a previous study, we explored the optimal parameters for synthesizing iron oxide nanoparticles using a microwave process, guided by an experimental design [30,40]. 

**Figure 1:**
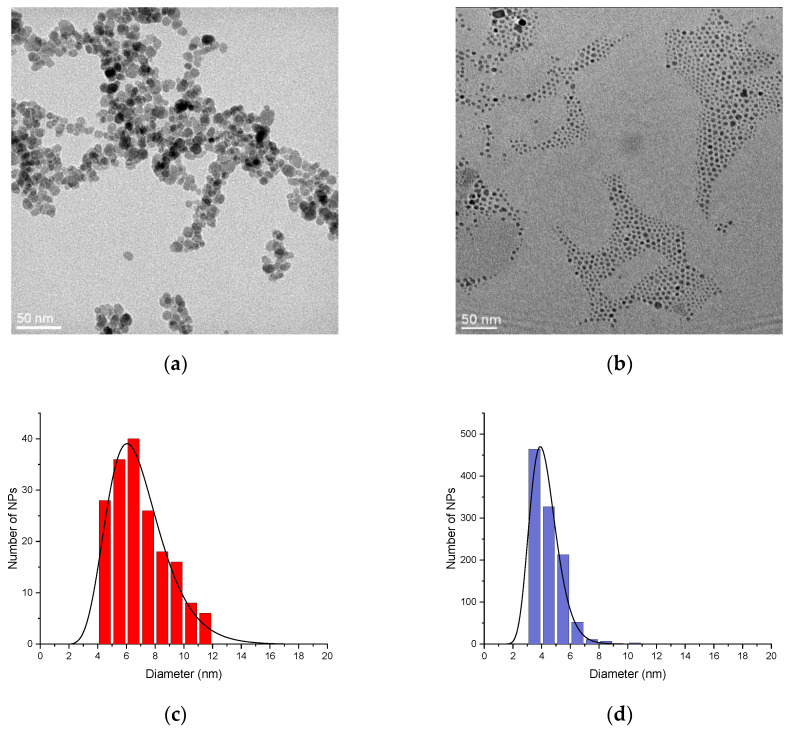
TEM images (**a**,**b**) and the size distribution (with a log-normal fitting) (**c**,**d**) of NPs_CoP_@Cit (red) and NPs_MW_@Cit (blue) respectively.

In the current study, we compare a sample synthesized under these optimal microwave conditions with a sample of iron oxide nanoparticles obtained through a conventional co-precipitation method, using identical experimental conditions. First, a comparison of the TEM micrographs is performed. The TEM micrograph in Figure 1a corresponds to the sample obtained by conventional co-precipitation (referred to as NPs_CoP_@Cit), while Figure 1b shows the sample synthesized via the microwave process (referred to as NPs_MW_@Cit). 

A noticeable difference between these two samples can be observed. The NPs_CoP_@Cit nanoparticles exhibit slight aggregation and vary in size, ranging from 4 nm to 12 nm (Figure 1c), with an average size of 6.6 ± 1.9 nm. In contrast, NPs_MW_@Cit nanoparticles are more uniform in size, ranging from 3 to 7 nm, with minimal aggregation (Figure 1d). The average size of NPs_MW_@Cit is 4.1 ± 0.8 nm. The microwave process produces nanoparticles that are more dispersed, free from aggregation, and uniform in size. Indeed, the standard deviation of NPs_MW_@Cit is half that of the conventional process, indicating that microwave synthesis enhances the monodispersity of the nanoparticles. To further confirm these findings, DLS measurements were conducted. 

**Figure 2:**
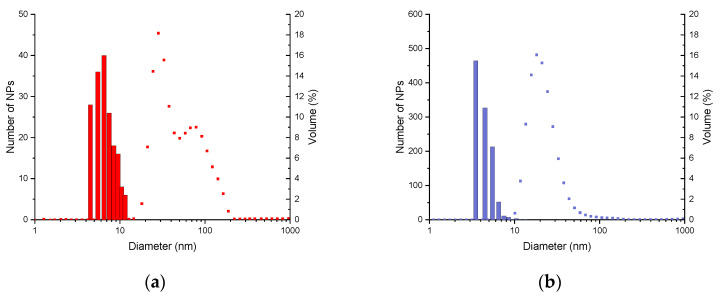
DLS measurements (point) in comparison with the size distribution obtained by TEM images: (**a**) of NPs_CoP_@Cit (red); (**b**) of NPs_MW_@Cit (blue).

The DLS measurements (Figure 2) allow us to determine the hydrodynamic diameter, which includes the diameter of the inorganic core, the organic layer, and the solvation sphere. For NPs_CoP_@Cit, two distinct populations of nanoparticles are observed, with average hydrodynamic diameters of 32 ± 8 nm and 102 ± 34 nm (Figure 2a). These two different hydrodynamic diameters confirm the presence of nanoparticle aggregation. In contrast, NPs_MW_@Cit exhibits a single hydrodynamic diameter of 23 ± 10 nm, which is smaller than that of NPs_CoP_@Cit. This smaller diameter, along with the presence of a single peak (Figure 2b), suggests either a smaller nanoparticle size or the absence of aggregation. Thus, the use of microwaves provides better control over nanoparticle size and dispersion.

**Figure 3:**
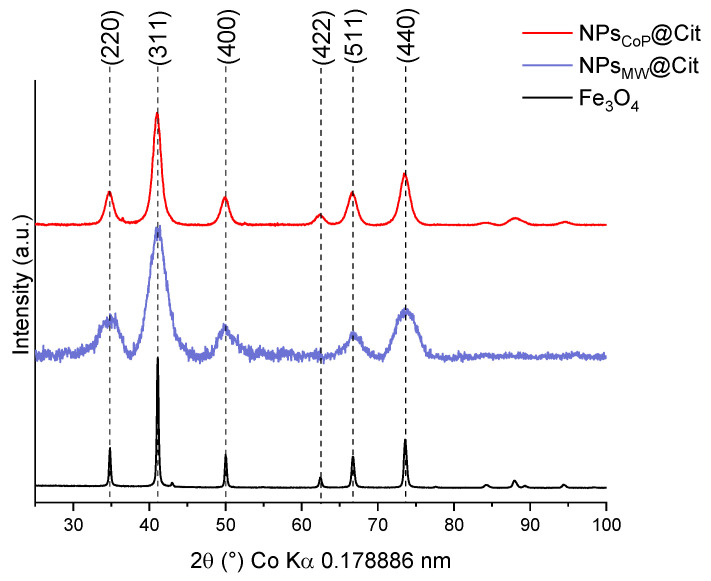
XRD measurements of NPs_CoP_@Cit (red), NPs_MW_@Cit (blue), and a reference of pure magnetite Fe_3_O_4_ (black).

To characterize the inorganic core of these nanoparticles, XRD measurements were performed (Figure 3). A magnetite reference (Fe_3_O_4_) was compared with the XRD patterns of NPs_CoP_@Cit and NPs_MW_@Cit to determine whether the inorganic core is magnetite or another iron oxide phase, such as hematite (α-Fe_2_O_3_) or maghemite (γ-Fe_2_O_3_). This reference sample was synthesized using the same protocol as NPs_CoP_@Cit, but without the ligand and under a controlled atmosphere to ensure the formation of pure magnetite.

Magnetite crystallizes in a cubic structure with the space group F_d$\bar{3}$m_, which is the same space group that maghemite (γ-Fe_2_O_3_) crystallizes in. The primary distinction between these two phases lies in their lattice parameters; due to the presence of vacancies in maghemite, its lattice parameter is slightly smaller than that of magnetite (8.354 Å for maghemite compared to 8.395 Å for magnetite) [6,41].

For both, the mean peaks of magnetite (indexing by the JCPDS card 04-015-8200) are present and for NPs_CoP_@Cit, a better crystallinity is present due to the aggregation of NPs. 

The lattice parameter was calculated using the average lattice parameter from each magnetite peak. For NPs_CoP_@Cit, the lattice parameter is 8.393 ± 0.014 Å, while for NPs_MW_@Cit, it is 8.389 ± 0.010 Å. Both values are close to the lattice parameter of bulk magnetite Fe_3_O_4_, which is 8.395 Å.

Using the Debye–Scherrer formula, the crystallite size was also calculated. For NPs_CoP_@Cit, the crystallite size is approximately 7.8 nm, which aligns with the diameter measured from TEM images (6.4 nm). Similarly, for NPs_MW_@Cit, the crystallite size is about 4.4 nm, which corresponds closely to the diameter obtained from TEM images (4.0 nm).

**Figure 4:**
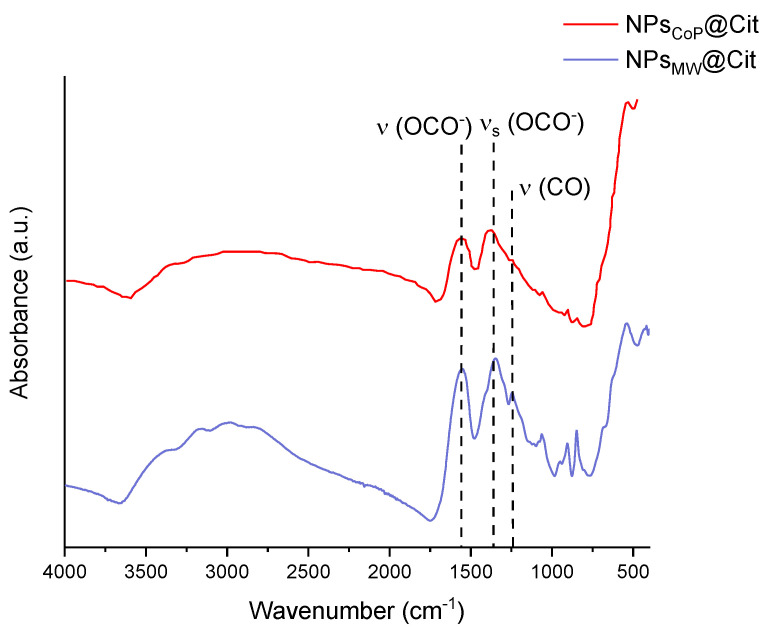
FTIR measurements of NPs_CoP_@Cit (red) and NPs_MW_@Cit (blue) with the position of some vibrational bands with black dotted lines.

The FTIR spectra of NPs_CoP_@Cit and NPs_MW_@Cit are presented in Figure 4. This technique allows for the identification of the ligands present on the surface of the nanoparticles, as well as the determination of the oxidation state of the iron atoms. 

The spectra of NPs_CoP_@Cit and NPs_MW_@Cit are identical, both featuring a broad, poorly resolved band from 3500 to 3000 cm^−1^. This band corresponds to the ν(OH^−^) stretch of hydroxyl groups from the citrates and physiosorbed water. The bands at 1560 and 1385 cm^−1^ are attributed to the asymmetric and symmetric stretching (OCO^−^) of the carboxylate groups from the ligands. The presence of these two bands confirms that the iron oxide nanoparticles are functionalized with citrates. Additionally, a vibrational band around 1260 cm^−1^ corresponds to the C-O stretching.

Iron can exist in various crystalline states, including magnetite (Fe_3_O_4_), maghemite (γ-Fe_2_O_3_), and hematite (α-Fe_2_O_3_). In this FTIR study, there was no control of the reaction atmosphere to prevent oxidation, making maghemite the more likely phase for the iron oxide nanoparticles synthesized in both samples, given the nanoparticle sizes determined by TEM. Both samples have an iron oxide core smaller than 8 nm, and previous studies suggest that such nanoparticles are typically composed of maghemite. The citrate functionalization during synthesis might have protected the nanoparticles from oxidation, leading to the formation of very small particles consisting of a mixture of maghemite and magnetite. The results also indicate that the basic nature of the precipitation process influences the nanoparticle composition. A single band around 580 cm^−1^ suggests a magnetite core, while a succession of bands from 500 to 800 cm^−1^ indicates a maghemite core (this band pattern is due to the presence of vacancies) [42]. For both samples, only one band is present around 580 cm^−1^, confirming the predominant presence of magnetite, with the citrate functionalization protecting against the oxidation of magnetite into maghemite.

**Figure A1:**
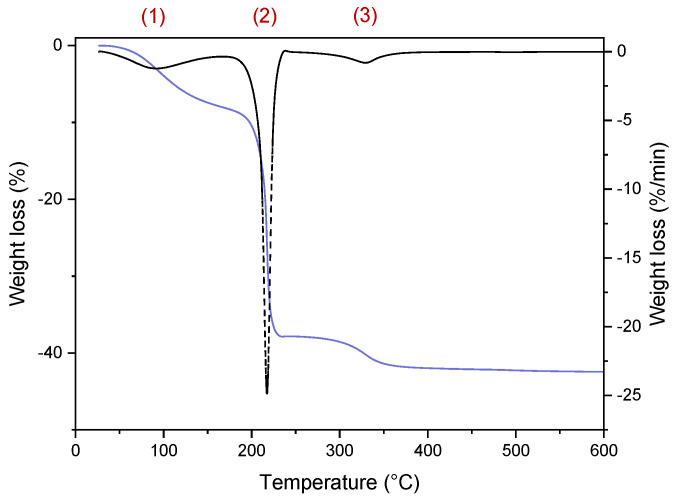
TGA curve of NPs_MW_@Cit from room temperature to 600 °C (blue line) with associated dTG (black dash) with a ramp rate of 10 °C/min to determine weight loss.

Subsequently, a TGA measurement (an example shown in Figure A1) was conducted to determine the iron oxide content and the ligand ratio in NPs_MW_@Cit and NPs_CoP_@Cit. The sample was heated to 600 °C to decompose the citrate ligands. The first weight loss, around 10%, occurred between 50 °C and 150 °C, corresponding to the evaporation of physiosorbed water (1 in Figure A1). The second stage, occurring at 200 °C, involved the most significant decomposition of the ligands, where the carboxylate groups decomposed into CO_2_ (2 in Figure A1). The final stage began at 330 °C, corresponding to the decomposition of the remaining chains (3 in Figure A1). The final weight was used to calculate the iron content, which was 58% for NPs_MW_@Cit and 69% for NPs_CoP_@Cit.

Iron oxides are particularly interesting in terms of magnetism due to their crystal lattice. Unlike hematite (α-Fe_2_O_3_), magnetite and maghemite are ferrimagnetic above a certain size, attributed to the proportion of Fe^2+^ and the vacancies in their crystal lattice. Nanoparticles smaller than 20 nm exhibit superparamagnetic behavior [6], meaning they have no magnetization at room temperature. In this state, the inherent energy of the nanoparticles is lower than the thermal energy, allowing them to spontaneously reverse their magnetic orientation. 

**Figure 5:**
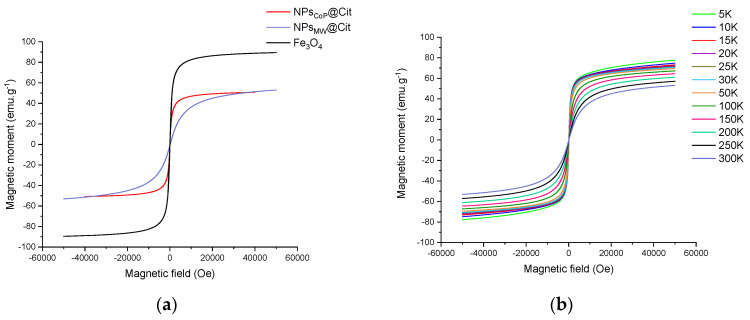
Magnetization as a function of magnetic field: (**a**) at room temperature (300 K) for NPs_CoP_@Cit (red), NPs_MW_@Cit (blue) and a reference of magnetite Fe_3_O_4_ (black); (**b**) at different temperatures (from 5 K to 300 K) of NPs_MW_@Cit.

The magnetization curve was measured between −50,000 Oe (10,000 Oe = 1 T) and 50,000 Oe at 300 K (Figure 5a). Both NPs_MW_@Cit and NPs_CoP_@Cit exhibited no hysteresis, indicating superparamagnetic behavior. The magnetization saturation (M_S_) was 53 emu.g^−1^ and 51 emu.g^−1^, respectively, showing similar values for both synthesis methods. In a superparamagnetic state at room temperature, the M_S_ value increases as the temperature decreases because the magnetic moments become locked. For NPs_MW_@Cit, this phenomenon was observed, with M_S_ increasing from 53 emu.g^−1^ at 300 K to 78 emu.g^−1^ at 5 K (Figure 5b).

**Figure 6:**
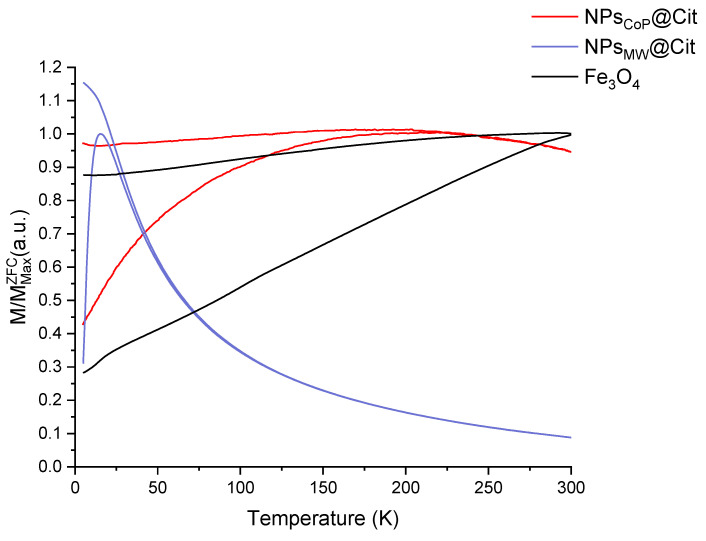
ZFC/FC curves of NPs_CoP_@Cit (red), NPs_MW_@Cit (blue) and a reference of magnetite Fe_3_O_4_ (black).

To confirm the superparamagnetic nature of NPs_MW_@Cit and NPs_CoP_@Cit, a Zero-Field-Cooled/Field-Cooled (ZFC/FC) measurement was conducted (Figure 6). This measurement provides crucial information about the blocking temperature (T_B_), which corresponds to the maximum of the ZFC curve. For NPs_MW_@Cit, T_B_ is 15 K, a temperature that correlates with the small size of the nanoparticles, indicating a very low blocking temperature. In contrast, for NPs_CoP_@Cit, T_B_ is 169 K, which aligns with the larger size and the aggregation of the nanoparticles produced by the co-precipitation method. 

## 3. Discussion

In this study, iron oxide nanoparticles were synthesized using two different methods: conventional co-precipitation (referred to as NPs_CoP_@Cit) and a microwave process (referred to as NPs_MW_@Cit). These two synthesis methods were then compared with a reference sample of iron oxide nanoparticles (Fe_3_O_4_) that was synthesized without an organic layer and under controlled atmospheric conditions. The synthesis parameters for both samples were selected based on findings from previous studies [30,40].

With conventional co-precipitation synthesis, NPs obtained have a little aggregation, but it is minimized due to the presence of the citrate layer [40]. However, to further reduce this aggregation and in addition to increase the monodispersity, a microwave process is carried out. Indeed, the heating in this synthesis is realized by the molecular vibration of the molecule of water (the solvent), so the heating is more homogeneous compare a classical heating [36]: there is not a temperature distribution inside the reactor. Due to this homogeneity of the temperature, there is a control of the size and the shape of NPs because the growth of each nanoparticle is dependent of the temperature. This homogeneity is translated by the reduction in the standard deviation for the size between NPs_CoP_@Cit and NPs_MW_@Cit (from 1.9 to 0.8). 

Moreover, this homogeneity is confirmed by DLS measurements. For the NPs_CoP_@Cit sample, two distinct peaks in hydrodynamic diameter were observed, corresponding to the combined size of the inorganic core, organic layer, and solvation sphere. The first peak, around 32 nm, represents the hydrodynamic diameter of isolated nanoparticles, while the second, around 102 nm, likely indicates the presence of aggregated nanoparticles. In contrast, NPs_MW_@Cit exhibits a single peak around 23 nm with a narrow distribution, further confirming the homogeneity of the nanoparticles in the solution.

These characterizations (TEM and DLS) yield consistent nanoparticle sizes, which are further corroborated by XRD analysis. Using the Debye–Scherrer equation, the crystallite size of the nanoparticles was calculated. Given the very small size of the NPs (below 20 nm), a modified formula was applied, with the crystallite size determined from the average of several main peaks of iron oxide nanoparticles. Due to the small size of NPs_CoP_@Cit and NPs_MW_@Cit, it is reasonable to assume that the crystallite size corresponds to the nanoparticle size. The calculated crystallite size aligns closely with the sizes obtained from TEM images: 7.8 nm for NPs_CoP_@Cit (TEM size: 6.4 nm) and 4.4 nm for NPs_MW_@Cit (TEM size: 4.0 nm). Additionally, the XRD pattern of NPs_CoP_@Cit is more sharply defined than that of NPs_MW_@Cit, which can be attributed to the larger nanoparticle size and the presence of aggregation. Aggregation leads to a more uniform behavior among nanoparticles, thereby enhancing the definition of the XRD pattern.

Finally, XRD measurements also allow for the calculation of the lattice parameter of the nanoparticles, which can be compared with a reference magnetite (Fe_3_O_4_). Pure magnetite has a lattice parameter of 8.395 Å, while pure maghemite (γ-Fe_2_O_3_) has a lattice parameter of 8.354 Å. In this study, the lattice parameters of NPs_CoP_@Cit and NPs_MW_@Cit were found to be 8.389 Å and 8.393 Å, respectively, indicating that the inorganic cores of both samples are composed of sub-stoichiometric magnetite (Fe_3−δ_O_4_).

After confirming the nature of the inorganic core, it is crucial to verify the presence of the organic layer. An initial indication of this organic layer is provided by DLS measurements, which describe the hydrodynamic diameter, encompassing the inorganic core, organic layer, and solvation sphere. The larger diameter obtained from DLS compared to TEM measurements suggests the presence of an organic layer. To conclusively confirm this, FTIR spectroscopy is utilized. The organic compound used to enhance the monodispersity of iron oxide nanoparticles, prevent aggregation, and solubilize the NPs in an aqueous solution is citrate. Citrate molecules possess three carboxylic groups (which are deprotonated due to the neutral pH of the solution), an alcohol group, and a methyl group. At least one carboxylate group binds the citrate molecules to the nanoparticle surface. In the FTIR spectra, the presence of the carboxylate group is indicated by two bands corresponding to the symmetric and asymmetric stretching of -OCO, located at 1385 and 1560 cm^−1^, respectively. The slight shift from their theoretical values of 1395 and 1590 cm^−1^ suggests an interaction between the ligands and the inorganic core [42]. 

Finally, the magnetic properties of the nanoparticles were analyzed through various magnetic characterizations. Due to their nanoscale size, the magnetic behavior of the nanoparticles differs from that of bulk materials. For iron oxide nanoparticles, particularly magnetite and maghemite, particles larger than 20 nm typically exhibit ferrimagnetic behavior. However, when the particle size is below 20 nm, they display superparamagnetic behavior, characterized by the absence of magnetization at room temperature. In this study, both NPs synthesized via conventional co-precipitation and the microwave process exhibited superparamagnetic behavior, confirming their size is below 20 nm. Furthermore, the magnetization saturation values (i.e., the maximum magnetic moment at a high magnetic field) for NPs_CoP_@Cit and NPs_MW_@Cit are closely aligned, at 51 and 53 emu.g^−1^, respectively, consistent with iron oxide nanoparticles of similar sizes.

To further confirm the superparamagnetic state, ZFC/FC (Zero Field Cooled/Field Cooled) measurements were conducted. These curves help determine the temperature at which the transition from a superparamagnetic to a blocked state occurs. This transition temperature corresponds to the peak of the ZFC curve [43]. For NPs_CoP_@Cit, the blocking temperature (T_B_) was found to be 169 K, a value consistent with the observed aggregation and size of the nanoparticles. In contrast, the T_B_ for NPs_MW_@Cit was significantly lower, at 15 K. The ZFC curve for NPs_MW_@Cit also showed a very narrow peak, indicating a narrow distribution of temperature transitions, which suggests that the nanoparticles are uniform in size. In the FC curve, magnetization increased continuously below T_B_, suggesting weakly interacting or isolated nanoparticles within the sample [43]. Consequently, citrate-functionalized nanoparticles with a microwave process were not aggregated in agreement with TEM results.

## 4. Materials and Methods

### 4.1. Synthesis of Nanoparticles by Co-Precipitation 

A mixture of iron (II) chloride tetrahydrate (FeCl_2_·4H_2_O, 5.03 mmol; CAS: 13478-10-9) and iron (III) chloride hexahydrate (FeCl_3_·6H_2_O, 8.88 mmol; CAS: 10025-77-1) was dissolved in 40 mL of ultrapure water with continuous magnetic stirring. Once the precursors were fully dissolved, citric acid (6.95 mmol; CAS: 77-92-9) in 5 mL of ultrapure water was added to the mixture. The solution was then heated to 80 °C using a water bath. Subsequently, 10 mL of ammonium hydroxide (NH_4_OH; CAS: 1336-21-6) was introduced to the mixture, inducing precipitation of the nanoparticles. These nanoparticles were designated as NPs_CoP_@Cit.

The washing process involved centrifuging the nanoparticle mixture with absolute ethanol at 10,000 rpm for 5 min, followed by removal of the supernatant. A subsequent washing step was performed using a combination of absolute ethanol and ultrapure water, again at 10,000 rpm for another 5 min. The resulting nanoparticles were then redispersed in ultrapure water.

### 4.2. Synthesis of Nanoparticles by Microwave-Assisted Co-Precipitation

The experimental conditions for the microwave synthesis mirrored those of the co-precipitation method, albeit with smaller volumes of materials. A mixture of FeCl_2_·4H_2_O (5.03 mmol; CAS 13478-10-9), FeCl_3_·6H_2_O (3.70 mmol; CAS 10025-77-1), citric acid (3.16 mmol; CAS 77-92-9), and 15 mL of ultrapure water was stirred until fully homogenized. Following this, 5 mL of ammonium hydroxide (NH_4_OH; CAS 1336-21-6) was added to precipitate the solution, which then turned black. The mixture was stirred again before being placed in a microwave reactor. The solution was heated to 96 °C for 40 min with continuous magnetic stirring (10 min ramping up to 96 °C, followed by 30 min at that temperature) and then allowed to cool down to 60 °C. The resulting nanoparticles were designated as NPs_MW_@Cit.

The washing process was identical to that used in the co-precipitation synthesis. It involved centrifuging the nanoparticle mixture with absolute ethanol at 10,000 rpm for 5 min, followed by removal of the supernatant. A subsequent washing step was performed using a combination of absolute ethanol and ultrapure water, again at 10,000 rpm for another 5 min. The final nanoparticles were then dispersed in ultrapure water.

### 4.3. Characterizations of Nanoparticles

#### 4.3.1. Transmission Electron Microscopy

Transmission electron microscopy (TEM) used for nanoparticles was a CM200—FEI operating at 200 kW (point resolution 0.27 nm) at different magnifications. The size distribution was calculated thanks to a free software ImageJ (Version 1.31) [44]. 

#### 4.3.2. Dynamic Light Scattering 

Dynamic light scattering (DLS) measurements were carried out using a ZETASIZER Nano ZS from Malvern Instruments. The nanoparticle samples in water were first diluted 100-fold to minimize electrostatic interactions and ensure accurate Brownian motion analysis [45]. This dilution was consistent with that used in TEM analysis. The DLS results can be expressed in terms of intensity, number, or volume distribution. In this study, volume distribution was chosen to investigate potential nanoparticle aggregation and determine the hydrodynamic diameter. The hydrodynamic diameter includes the diameter of the inorganic core, the organic layer, and the solvation sphere.

#### 4.3.3. X-ray Diffraction

X-ray diffraction (XRD) pattern was recorded at room temperature with an INEL CPS120 equipped with a monochromatic cobalt radiation (Co Kα = 0.178886 nm) at grazing angle of incidence simultaneously on 120°. 

#### 4.3.4. Fourier Transform Infrared Spectroscopy

Fourier Transform Infrared Spectroscopy (FTIR) was performed with a Nicolet 6700 (Thermo Fisher, Waltham, MA, USA) from 400 cm^−1^ to 4000 cm^−1^ in absorbance. 

#### 4.3.5. Thermogravimetric Analysis

Thermogravimetric analysis (TGA) was carried out on a nanoparticle’s sample dried by a heat chamber. The device used was a microbalance SETSYS EV 1750 TGA from the brand named Setaram. The measurement consisted of a defined mass of the sample in a crucible and increase in temperature from 20 to 600 °C at 10 °C/min under air flow. At the end, the exact mass of iron oxide in the sample was given. 

#### 4.3.6. Magnetic Measurement 

Magnetic measurements were conducted on dried nanoparticle powder. The nanoparticles were placed on a glass sample holder and secured with KAPTON adhesive tape to prevent any movement. The measurements were taken using a SQUID (Superconducting Quantum Interference Device) equipped with a VSM (Vibrating Sample Magnetometer) head from Quantum Design. This device was used to determine the magnetization saturation (M_S_) at both room temperature (300 K) and low temperature (5 K) by applying a magnetic field cycle from +50,000 Oe (1 T = 10,000 Oe) to −50,000 Oe, and back from −50,000 Oe to +50,000 Oe.

To determine the blocking temperature (T_B_), the transition temperature between a blocked state and a superparamagnetic state, Zero Field Cooled (ZFC) and Field Cooled (FC) measurements were performed. Initially, the sample was cooled to 5 K without an applied magnetic field. Then, a magnetic field of 200 Oe was applied, and the sample was heated from 5 K to 300 K while the magnetization was recorded, resulting in the ZFC curve. Subsequently, under the same magnetic field, the sample was cooled from 300 K back to 5 K, with the magnetization recorded to generate the FC curve. The T_B_ value is associated with the peak of the ZFC curve. However, this determination can be imprecise due to the distribution of blocking temperatures, with Monte Carlo simulations offering a more accurate method for determining T_B_ [43]. But, for practical reasons, the maximum of the ZFC curve is used.

## 5. Conclusions

In this study, we developed a rapid, one-step method for synthesizing citrate-functionalized iron oxide nanoparticles (NPs) using microwave technology. The iron oxide NPs produced through this microwave-assisted synthesis are monodispersed, exhibiting excellent colloidal stability with an average size of 4.1 ± 0.8 nm. These NPs were also compared with those synthesized via the conventional co-precipitation method. The results show that NPs synthesized using microwaves are better dispersed and have a narrower size distribution than those obtained from co-precipitation. Moreover, aggregation of NPs is more pronounced in the co-precipitation method compared to the microwave method, indicating that microwave synthesis enhances the monodispersity of NPs.

Importantly, the use of microwaves does not compromise the physicochemical properties of the NPs. This method allows for the rapid production of iron oxide nanoparticles that are free from aggregation and exhibit good crystallinity. Additionally, we confirmed the superparamagnetic nature of these microwave-synthesized NPs, making them strong candidates for biomedical applications, such as MRI contrast agents.

## Figures and Tables

**Table 1:** Summarize of the parameters, advantages and disadvantages of different iron oxide nanoparticle synthesis.

Synthesis	Conditions	T (°C)	Time	Solvent	Size of NPs (nm)	Size Distribution	Advantages	Disadvantages
Hydrothermal	Simple	>200	Hours	Water	<1000	Narrow	Easy control of the size and shape	Synthesis with high pressure and temperature
Sonochemistry	Simple	30–50	Several minutes to hours	Water	<100	Narrow	Control of the size	Complete mechanism still unknown
Sol–gel process	Complex	20–100	Hours	Water	6–15	Good	Control of the size and shape	Low wear resistance
Thermal decomposition	Complex	200–400	Hours	Organic solvent	<20	Narrow	Total control of the size and shape	Synthesis at high temperature and with organic solvent
Co-precipitation	Simple	20–100	Minutes to few hours	Water	<20	Good to large	Easy, quickly and with a huge yield	No control of the shape/size. Formation of aggregation
Microwave process	Simple	30–120	Minutes	Water	<20	Narrow	Easy, quickly, control of the size	Low yield (due to the volume of the reactor)

## Data Availability

Data are contained within the article.

## Appendix A

Example of TGA curve of iron oxide nanoparticles from room temperature to 600 °C.
